# A Middle Eastern journey of integrating Interprofessional Education into the healthcare curriculum: a SWOC analysis

**DOI:** 10.1186/s12909-016-0852-5

**Published:** 2017-01-17

**Authors:** Alla El-Awaisi, Kyle John Wilby, Kerry Wilbur, Maguy Saffouh El Hajj, Ahmed Awaisu, Bridget Paravattil

**Affiliations:** College of Pharmacy, Qatar University, Doha, Qatar

**Keywords:** Interprofessional education, SWOC analysis, Pharmacy, Middle East, Curriculum

## Abstract

**Background:**

Interprofessional education (IPE) is an emerging concept in the Middle East with a number of health professional degree programs continually striving to meet international accreditation requirements to enhance the quality of education and ensure high standards are maintained. Using the College of Pharmacy at Qatar University (CPH QU) as a model, this article describes the IPE initiatives coordinated through the College’s IPE Committee, with representation from fourteen programs at four Healthcare institutions: Qatar University; Weill Cornell Medical College in Qatar; the University of Calgary in Qatar; and the College of North Atlantic in Qatar. These activities are based on the model proposed by the University of British Columbia across the different pharmacy professional years. Learning objectives for these initiatives were selected from the IPE shared competency domains and competency statements developed for Qatar context.

**Method:**

A meeting with six faculty members, who have been instrumental to designing and executing the IPE activities in the previous 2 years, was convened. Faculty members reflected on IPE activities and collaborations with other participating programs. A structured SWOC (Strengths, Weaknesses, Opportunities, Challenges) framework was used to guide discussion. The discussion was recorded and notes were taken during the meeting. Raised points were categorized into each SWOC category for the final analysis.

**Results:**

Implementation of IPE program is a major undertaking with a number of challenges that require invested time to overcome. This article highlights the importance of incorporating IPE into healthcare curricula to graduate students ready for collaborative practice in the workforce. Learning objectives for IPE initiatives need to be based on shared competency domains. When developing and implementing an IPE program it is necessary to align activities under a strong theoretical framework. This should be done under the leadership of an IPE steering group or committee to oversee the integration of IPE into the healthcare curriculum.

**Conclusion:**

The article presents many lessons learned through IPE implementation that are relevant to other academic institutions keen to incorporate IPE into their programs and also provides a successful model for integrating IPE into healthcare curricula.

## Background

Interprofessional education (IPE) provides opportunities for healthcare professional students to learn with, from and about each other to improve collaboration and the quality of patient care upon graduation [1]. For more than a decade, the integration of IPE into healthcare curricula has been embraced to enrich students’ learning experiences in ways to prepare them for a collaborative practice environment, where professional silos are dissolved and effective collaborative and non-hierarchical relationships are enhanced and nurtured [2]. The World Health Organization (WHO) Framework for Action on Interprofessional Education and Collaborative Practice highlights the vital role of incorporating IPE into healthcare curricula to create and prepare a healthcare workforce best able to meet the changing and complex health challenges facing today’s world [3]. It also emphasizes that an effective model of interprofessional collaboration should be regionally distinct, taking into account the unique needs and sensitivities of particular environments while striving to maintain the highest standards of care.

IPE is an emerging concept in the Middle East with a number of health professional degree programs continually striving to meet international accreditation requirements to enhance the quality of education and ensure high standards are maintained. Taking the State of Qatar as an example and through its National Vision 2030 (government mandate), it aims to establish a comprehensive world-class integrated healthcare system to improve the health of Qatar’s population and provide them with best quality care delivered by a highly skilled healthcare workforce [4]. Adoption of IPE among Qatar health professional training programs is therefore aligned with local needs and international standards. The purpose of this article is to: (1) describe how IPE has been developed and integrated into the four professional years of pharmacy curricula at Qatar University (QU) College of Pharmacy (CPH) with collaboration from all healthcare education programs within the university and institutions within the country and; (2) identify the strengths, weaknesses, opportunities and challenges (SWOC) related to the integration of IPE into the pharmacy curriculum through reflection and discussion among key stakeholders.

### How we developed, integrated, and implemented IPE in Qatar

The Bachelor of Pharmacy degree program at QU CPH is the first outside of Canada to be fully accredited by the Canadian Council for Accreditation of Pharmacy Programs (CCAPP). Since January 2013, CCAPP accreditation standards have outlined the need for programs to provide IPE experiences for pharmacy students and demonstrate evidence of how these opportunities are executed [5]. Consequently, an IPE Committee (IPEC) was established at QU CPH in 2014 to provide guidance and support in implementing IPE within the pharmacy curriculum, as well as in our partner healthcare training programs in the country. Committee members include representatives from fourteen programs at four institutions: (1) QU: CPH, College of Medicine (CMED) and the newly formed College of Health Sciences (CHS) (previously known as Department of Health Science: Biomedical Science, Public Health, Human Nutrition) and Sports Science Program at the College of Arts and Science (CAS); (2) Weill Cornell Medical College in Qatar (WCMC-Q); (3) the University of Calgary – Qatar (UC-Q) nursing school and; (4) the College of North Atlantic Qatar (CNA-Q): dental assistant, advanced care paramedicine, environmental health, medical radiography technology, pharmacy technician, and respiratory therapy programs.

### The Model of IPE adopted

When developing and delivering an IPE program at any level (institutional, regional, national), it is necessary to align activities under a theoretical framework that allows for coordinated design and implementation in terms of objectives, content, complexity, and delivery. Miller’s pyramid of clinical competence was used to guide the objectives of our activities [6]. This theory proposes that learners move from a knowledge phase (knows and knows how), through a demonstration phase (shows how), and finally to a competent phase of ‘doing’. We believe IPE to be a continuum of competence that requires students to progress through these phases in order to adequately provide care in interprofessional settings. It is our aim to ensure students learn (know how), demonstrate (show how), and finally perform effectively in these settings upon completion of the pharmacy program.

Miller’s theory was used to inform session objectives but we still required a theoretical model to guide the content, complexity and delivery of IPE activities. To address this, we have adapted a model proposed by the University of British Columbia which accounts for differences in student readiness to learn within interprofessional settings, as well as their learning needs at various times throughout the learning process [7]. The model is based on three key concepts: Exposure, Immersion, and Mastery. The theoretical model allows for coordination of IPE activities to student learning needs at a given point in time in their training and compliments the behavioural approach of Miller’s model. In students’ early years in health professional programs, “exposure” activities (introduction of concepts, role clarification, student society activities etc.) are meant to promote learners understanding of their own roles and those of other professions within the healthcare setting. “Immersion” activities build upon the knowledge and confidence obtained during exposure activities and promote self-reflection, through higher level of interaction, collaboration and shared decision making with healthcare students, to enable transformation of student views regarding the role of themselves and others, with a desired outcome to have students acquire an ‘interprofessional world view’. Finally, the “mastery” stage strives to have senior students and graduates develop an advanced level of critical thinking, high degree of self-reflection, and greater understanding of differing professional roles and contributions, ideally within actual healthcare settings. Those achieving this stage are able to implement interprofessional teamwork in practice and also teach concepts of collaboration to others [7].

### The IPE initiatives and activities implemented

Table 1 describes the IPE initiatives coordinated through IPEC across the different pharmacy professional years, according to the above model. These IPE activities incorporated 2–6 different professions and lasted between 2–3 h each. Activities were held at different campuses. Learning outcomes were selected from IPE shared competency domains and statements previously created for Qatar by healthcare professional educators, which included: professional-role clarification; interprofessional communication; patient-centered care; and shared decision-making [8]. During the IPE activities, the large group is divided into smaller groups ensuring all the participating healthcare professions are represented. A nominated spokesperson from each small group is always encouraged to present their learning findings to the larger group, an exercise encouraging recognition of collaboration necessary among healthcare disciplines to optimize patient care [9]. Time is always dedicated at the beginning of each IPE introductory session describing the IPE activity, getting to know each other exploring each other’s discipline, followed by an icebreaker game.

**Table 1:** Interprofessional Education Activities per each Pharmacy Professional Year (Academic Year AY13-AY15)

Evidence/Behavior (Miller 1990)	Model of IPE (UBC 2009)	IPE Activity	Description of IPE Activity	Assessment
First Professional Year				
Knows How	E	Introducing IPE: • AY14: 52 students representing two professions: Pharmacy (QU), Human Nutrition (QU) and sport science (QU) students • AY15: 112 students representing four professions: Pharmacy (QU), Medicine (QU), Nursing (UCQ) and Human Nutrition (QU). • Two hour session	The session introduced students to the concept of IPE and team communication and started with an ice-breaker game. This was followed with two short videos highlighting interprofessional shared competencies and students were asked in their interprofessional teams to reflect on these and discuss their importance. The students then played a game where they had a series of ten professions and they had to match them to the correct role description. The student were then given a simple case about a patient recently diagnosed with diabetes and in their interprofessional teams they needed to identify which professionals would best meet the needs of this patient in the case, what would each profession do and how will the professions work together in a collaborative and effective team environment. Each activity ended with a large group summary discussion.	Student perceptions of team-based learning Material included on final course examination
Second Year Professional Year				
Knows How	E	IPE Smoking Cessation • AY14: 51 students representing four professions: Pharmacy (QU), Medicine (WCMC-Q), Pharmacy Technician (CNAQ) and Public Health (QU). • AY15: 72 students representing five professions: Medicine (WCMC-Q), Nursing (UCQ), Pharmacy (QU), Public Health (QU) and Respiratory Therapy (CNAQ) • Three hour session	This IPE activity started with a short introduction about the IPE activity, getting to know each other session in which students in groups were asked to explore each other’s discipline, followed an icebreaker game entitled ‘Common Ground Game’. A short didactic lecture on tobacco use treatment followed, highlighting the challenges encountered by smokers as well as non-pharmacologic and pharmacologic treatment modalities for smoking cessation. Students then watched two short videos on motivational interviewing. The students were then given a case scenario of a patient recently diagnosed with chronic obstructive pulmonary disease (COPD) who was a heavy smoker and desired to quit smoking. Students initially discussed each member’s professional perspective before making a team decision on the management of the case. Finally, students were asked to establish criteria for a smoking cessation clinic and consider how each discipline would work collaboratively to ensure delivered clinic care was patient-centered.	Student perceptions of team-based learning Group reflective IPE assignment
Knows How	E/I	Being An effective Team Player • AY13: 50 students representing three professions: Pharmacy (QU), Nursing (UCA) and Nutrition (QU). • AY14: 112 students representing six professions: pharmacy (QU), medicine (WCMC-Q), nursing (UCQ), pharmacy technician (CNAQ), medical radiography (CNAQ) and paramedicine (CNAQ). • AY15: 85 students representing six profession: Medicine (WCMC-Q), Nursing (UCQ), Paramedicine (CNAQ), Pharmacy (QU), Pharmacy Technician (CNAQ) and Respiratory Therapy (CNAQ). •Three hour session	This IPE activity focused on the importance of teamwork in patient safety. The IPE started with a short introduction defining the meaning of IPE and an ice breaker. The students in their groups watched a video exploring the concept of team communication and decision making and were asked to reflect on it by answering given questions and were encouraged to discuss their professional perspectives. The students were then given a case scenario of a patient recently admitted to the emergency department with a paracetamol overdose resulting in his death due to faulty communication by the team. They were asked to discuss the case and draw a diagram of the flow of information among the health professionals in this story and highlight the points of communication breakdown and discuss strategies that could have prevented this from happening.	Student perceptions of team-based learning
Knows How	E/I	Introduction to case-based integrated care planning: • AY13: 41 students representing two professions: Pharmacy (QU) and Human Nutrition (QU). • 2 h session	This IPE activity focused on introducing students to shared decision-making and team-based care. The session occurred over 2 h and no required pre-readings or preparation were given to students in advance. All students had taken respective therapy instruction on Crohn’s Disease before the session. After a brief introduction and overview of the session outline, students were formed into small groups, given a paper-based case that described a Crohn’s Disease patient, and were required to identify and solve drug and nutrition therapy problems. Students were expected to integrate patient-specific goals of therapy, therapeutic alternatives (both pharmacological and non-pharmacological) that could be recommended for the patient, a final recommendation with justification to support their decision, and a monitoring plan for both efficacy and safety. The last 30 min of the session were reserved for large group discussion, facilitated by faculty from each discipline. Discussion focused on student feedback for each care plan component, as well as IPE competencies such as role clarification and shared decision-making.	Student perceptions of team-based learning Material included on final course examination
Third Year Professional Year				
Shows How	I	Diabetes Case Based Patient Care • AY13: 33 students representing two professions: pharmacy (QU) and nursing (UCQ). • AY14: 72 students representing two professions: pharmacy (QU) and nursing (UCQ). • AY15: 85 students representing two professions: pharmacy (QU) and nursing (UCQ). • Three hour session	Course instructors adapted a diabetes case from an existing nursing resource to represent the progress of a patient through four stages of diabetic ketoacidosis (DKA): 1)diagnosis and initial emergency department management; 2) admission to a critical care unit; 3) transition to a general medicine ward; and finally 4) discharge planning for home. A short case synopsis was provided to the classes in advance. Initial development and delivery of this activity involved 20 pharmacy students joining 8 nursing students at the UC-Q campus for a 2-h session. Following the session orientation and “icebreaker” activity, four pre-assigned mixed-discipline groups were formed and in the 30 min allotted, students worked together to answer pathophysiology and pharmacology questions within their assigned DKA care stage and formulated and prioritized a management plan according to their assessment of patient data provided, requirements for medical management and anticipated follow-up monitoring needs. All members then convened for each group’s presentation of assessment and management plans followed by open class discussion and clarifications at 15-min intervals.	Student reflection Exercise DKA cases on final exam
Shows How	I	Diabetes Simulated Based Patient Care • AY14: 65 students representing two professions: pharmacy (QU) and medicine (WCMC-Q).	Course contributors developed two diabetes case content and standardized patient (SP) scripts: one ambulatory clinic visit and one in-patient admission. Students received disease-related pre-readings before the IPE activity. Eight facilitators participated in the session which opened with a joint introduction followed by breakout of the pre-determined mixed discipline groups each assigned to one of the two cases which were portrayed by WCMC-Q SPs trained by the course contributors. Student teams were provided a written summary containing the patient chief complaint, medical and medication history, and pertinent investigations. With this data, they had 15 min to discuss their initial assessment, each discipline’s roles, and arrive at a consensus for information gathering priorities when they met the patient. They next had 10 min to interview the SP who appeared at the simulated care environment, to confirm existing and acquire new information. At the conclusion of this interview, the teams then collaborated to propose a patient care plan. Facilitators who had observed the encounters then led a 20-min debriefing discussion regarding the team findings, management decision-making and shared-care experience.	Student reflection Exercise
Fourth Year Professional Year				
Shows How	I	Semester-long approach to integrated case-based learning: • AY14: 38 students representing two professions: Pharmacy (QU) and Human Nutrition (QU) • AY15: 51 students representing two professions: Pharmacy (QU) and Human Nutrition (QU) • Initial face-to-face session for ice breakers and case distribution • Care plan for case due 6 weeks later and release of new case • Conclusion event and submission of SOAP note 6 weeks later	This semester-long course-based IPE interaction focused on care planning and health record documentation for students in their final year of study. The first session was given at the start of the semester and focused on the importance of IPE in practice, an icebreaker activity, and instructions for the first assignment. Students were instructed to work together in pre-assigned groups (consisting of both pharmacy and nutrition students) to develop a comprehensive, integrated care plan according to a case that was pre-developed by session leaders. In this instance, the case focused on cardiovascular disease that required the students to assess both pharmacological and non-pharmacological approaches to care. Students were also asked to document the amount and types (face-to-face, telephone, email, videoconference, text message) of interactions during this stage of the activity. Approximately 2 months later, an integrated care plan was due for submission by student groups. Faculty from each respective program reviewed care plans and graded according to pre-established rubrics. Then, faculty updated the original case according to student answers and remotely instructed students to work within groups to solve the case and submit an integrated SOAP note (i.e. form of health record documentation) for evaluation. Upon completion of this portion, a large group face-to-face interaction occurred that summarized learning points and reviewed case answers. Learning objectives were assessed according to policies required from both programs.	Integrated Care Plan Integrated SOAP note Interaction log (types and amounts of student interaction) submitted not for credit
Knows How	I	Experiential Training • AY14 & AY15: 25 pharmacy students (QU) per academic year • Shadow & Interview Healthcare Professionals • Participation in IPE Educational Session	Two IPE activities were incorporated into the undergraduate experiential training for the 4th year pharmacy students. The first activity provided students the opportunity to learn about and from the healthcare members within the hospital setting. Students were required to shadow two healthcare professionals from different disciplines for a minimum of one hour and then interview them to understand their role and contribution as a team member. A series of suggested questions were provided to the student to facilitate the interviewing of these professionals. After completion of the first activity, each student provided a one page reflection on the IPE experience. The second IPE activity required the pharmacy student to participate in an interprofessional team educational session where 2 or more healthcare professionals were actively engaged in a teaching or discussion session. Students were provided with pre and post session reflection questions to facilitate writing an one page reflection on this educational session. These IPE activities were adapted from the Centre for Interprofessional Education at the University of Toronto.	Two graded reflection Assignment

## Methods

### The SWOC analysis

Since the inception of the undergraduate IPE curriculum, we have had more than 20 successful activities involving nine diverse groups of healthcare professional students including medicine, nursing, nutrition, pharmacy, pharmacy technician, public health, paramedicine, respiratory therapy and sports science from four different campuses. The IPEC advises and coordinates IPE at our College in cooperation with other healthcare discipline programs in Qatar. In an effort for continuous quality improvement of teaching innovations in our pharmacy degree program, we convened a meeting with six faculty members who have been instrumental to designing and executing IPE course content thus far to reflect on the respective activities in which they were involved. Participants were required to reflect in accordance with the domains of a SWOC analysis. The meeting was recorded and two faculty members also made notes throughout the session.

Participants made points regarding each domain of the SWOC matrix and consensus, by all the participating faculty, was a requirement for any point to be included in the analysis. In order to ensure the comprehensiveness of the data generated, discussion was stopped whenever it was deemed that the collection of new data does not shed any further light on the issue under discussion and that points are being duplicated. Through the method of consensus, the participants went over the list and removed any points that were interpreted as duplicates of others.

## Results

In this section, we present the results of the SWOC analysis in a narrative manner. A summary of the key points derived from the SWOC analysis are presented in Table 2.

**Table 2:** Results of SWOC Analysis

Strengths	Weaknesses
• Having motivated faculty members • Diversity of disciplines who are willing to participate in IPE • Existence of IPE Steering Committee that ensures quality control • Shared competencies that are tailored for Qatar • Motivated students and students perceptions determined through RIPLS and feedback • Output: successful implementation of several IPE activities • Output: peer-reviewed abstracts and scholarly publications • Mentorship to others who have less experience • International collaboration with experts in the field • Rapid development and speedy actions on new ideas	• Time allocated in curriculum may not be sufficient • Location of the different healthcare schools • Experiential training location and timing mismatch with IPE • Few professional development activities for those involved • Lack of adequate IPE training and experience • Lack of sufficient IPE facilitation experience • Lack of impact assessment on educational outcomes • Lack of funds and manpower dedicated for IPE • Lack of faculty recognition (CE points, workload) • Nonexistence of simulation labs and standardized patient (SP) programs
Opportunities	Challenges
• Leadership opportunity through organizing and hosting regional IPE conference • Establishment of IPE students society in the country • Assessment of impact on practice (collaborative care activities increasing in Qatar) • Provided a new area of research, scholarship and productivity • Meeting accreditation agency standards (Canadian Council on the Accreditation of Pharmacy Programs) • An opportunity to poster collaboration with a new College of Medicine • Compliance with and recognition from national regulatory bodies (Supreme Council of Health policies and regulations) • Use of relevant IPE activities to earn CE points • Plans to have a dedicated IPE center • An opportunity for students exchange	• Gender segregation due to cultural issues • Curriculum alignment is lacking from partners • Students buy-in on IPE philosophy • Managing workload due to lack of faculty designation and dedicated personnel • Discrepant efforts between different professions • Incorporation of all relevant disciplines in a particular IPE activity and case • Logistic difficulties are a significant challenge to IPE • Assessment of impact in real-life practice is a challenge • Sustainability of the program • Lack of structured and objective way of students’ assessment • Discrepancies in students number (e.g. too many medical students implies that they may dominate) and their levels of study • Current state in practice (collaborative practice) not matching with the educational developments

### Strengths

The SWOC analysis revealed some major strengths of our IPE curriculum. Most notably, there appears to be a large pool of highly motivated and dedicated faculty members and motivated students from across health professional training programs who are willing to embrace new educational initiatives in Qatar. The relatively large number of successfully designed and implemented IPE activities held over the last 2 years attest to this notion. Another identified strength is the diversity of healthcare professional disciplines in Qatar such as pharmacy, medicine, nursing, public health, nutrition, sports science, respiratory therapy, and others who have demonstrated willingness to actively participate in IPE and enthusiastic to ensure success. Furthermore, the IPE activities are based on shared competency domains and statements that were specifically developed for a Middle East context, ensuring cultural relevance. Finally, we have also developed ties with international partners with IPE expertise, i.e. UK-based Centre for Advancement of Interprofessional Education (CAIPE) and the Robert Gordon University in the UK, in order to build capacity and to enhance professional development and networking.

### Weaknesses

The identified weaknesses include: inadequate formal IPE and facilitation training, lack of sufficient professional development activities for novice faculty members involved in IPE, non-existence of impact assessment on educational outcomes (i.e. short- and long-term measures of success, specifically as it relates to collaborative care in practice and resultant patient outcomes), limited number of established simulation laboratories, and informal standardized patient (SP) programs at QU CPH.

### Opportunities

The introduction of IPE in the country has resulted in numerous opportunities for our programs and for QU CPH in particular. Not only are we leading national IPE course activity coordination through IPEC, but QU CPH also hosted the first Middle East regional IPE conference in December 2015 [10]. Furthermore, the IPE initiatives offer unprecedented opportunity to fulfill international accreditation standards and in Qatar. Lastly, the IPE has provided a new area of educational research, which has fostered pivotal networking and collaboration as well as scholarship in teaching and learning.

### Challenges

We identified several threats and challenges facing our IPE initiatives. These include, but are not limited to, gender segregation due to cultural barriers and policies (the college of pharmacy admits only female students to its undergraduate program), limited dedicated time to establish IPE activities for faculty members, discrepant efforts between different professions, logistic difficulties in planning and implementing IPE, lack of objective performance assessment methods, and difficulties in determining the impact of IPE in in real-world practice.

## Discussion

In the last few years, IPE has become an important element of teaching and learning in different healthcare disciplines and its diffusion is becoming increasingly important globally. IPE has turned into an essential component in training healthcare students and preparing them for effective collaborative practice, which in turn optimizes the delivery of healthcare services and potentially improves healthcare outcomes [3]. This paper is the first in the Middle East to report the pharmacy faculty members’ perceptions of areas of strengths, weaknesses, opportunities and challenges as they pertain to IPE implementation. It also highlights QU CPH leadership in successfully incorporating IPE within the healthcare curricula in Qatar.

Faculty members have highlighted several areas of perceived strengths, which have served as catalysts for IPE implementation in Qatar. One such attribute was engagement of highly motivated contributors eager to plan and facilitate IPE activities. A recent study exploring the awareness, views, and attitudes of pharmacy academics in Qatar towards IPE and collaborative practice have shown that QU CPH faculty members have positive attitudes toward IPE and perceive it to help healthcare professional students develop teamwork and communication skills [11]. These findings are very promising as negative faculty attitudes pose a significant challenge for designing and implementing IPE activities [12]. Another strength that was highlighted in the current study was the existence and leading role of IPEC at QU CPH. Since its inception, the committee has truly been the driving force for all IPE activities undertaken by pharmacy students at QU and otherwise [13]. Having existing core IPE competencies developed and shared across all Qatar health professional education programs was also mentioned as a strength [14]. These competencies serve as a guide to develop any IPE-related activity and to strategize teaching and assessment approaches. Collaboration with international IPE leaders was identified as another strength. Our pharmacy faculty built strong ties with Robert Gordon University (RGU) in the United Kingdom (UK), a pioneer in developing and integrating interprofessional learning across different healthcare programs [15]. Under this partnership, two RGU faculty conducted an IPE symposium in Qatar to provide local healthcare faculty members with the knowledge and skills necessary to improve IPE educational programs with a particular focus on facilitation skills [13].

In 2015, QU CPH hosted the first Middle East conference on Interprofessional Education, an event endorsed by CAIPE. This inaugural conference marked a new chapter for healthcare education not just in Qatar but also in the wider Middle East and North Africa region. Its goal was to equip attendees with the knowledge and confidence to implement IPE principles directly into their curriculum and to establish a network of skilled and innovative educators, dedicated to the principles of IPE. The conference was attended by over 300 healthcare academics, practitioners and health management leaders from 13 different countries including: Australia, Bahrain, Canada, Egypt, Iraq, Kuwait, Lebanon, Oman, Saudi Arabia, the United Arab Emirates, Turkey, the UK and United States. During the 3-day conference, there were six different workshops, 37 oral presentations and 40 posters displayed. IPE-related research output by QU CPH faculty members was also mentioned as another strength of our IPE [10, 13, 16–18]. In addition, availability of mentors for new faculty members was also perceived favourably. In fact, many healthcare programs have demonstrated that mentorship and coaching programs can offer effective support for faculty members. Mentorship by senior colleagues who had more opportunities to work in an interprofessional team can better prepare junior members to become future IPE champions [19].

One of the weaknesses identified in the SWOC analysis was the lack of IPE training and facilitation experience. This is a very common weakness or barrier for IPE implementation reported in many countries [20–22]. Bringing faculty members from different health care programs into the same classrooms would not necessarily lead to a successful IPE activity. Cross-discipline collaboration is to be encouraged among faculty members. Designing more IPE-related faculty development workshops may enhance the faculty members’ confidence and readiness to engage in IPE activities and may improve their capability to facilitate learning among diverse groups of students [12]. Other perceived weaknesses as faculty attempt to engage in IPE activities included high teaching workload, lack of adequate dedicated time for IPE and little or no recognition for faculty engagement in IPE. Effective IPE programs mandate reliable administrative and organizational support as IPE entails significant time and resources. It has been reported that IPE requires three times the preparation of other teaching activities [13]. In order to sustain our IPE initiatives, more support is needed from the administration of the different academic institutions and more protected time should be dedicated to IPE. Another identified weakness was the lack of sufficient information regarding the impact of our IPE activities on achieving the different student educational outcomes in relation to interprofessional care. More assessment studies looking at both short- and long-term outcomes of IPE activities including important impact on practice and resultant patient care are warranted and indeed, now strongly advocated by the Institute of Medicine [23]. The disparate location of participating academic institutions on five different campuses was also problematic. Qatar University has five health-oriented training programs on campus, but other key disciplines of nursing and medicine are elsewhere, often requiring an hour’s drive across the capital city. Ultimately, more faculty resources are devoted to organizing IPE across these campuses, including addressing logistical difficulties associated with student transport to and from any IPE event held outside their respective university.

The lack of formal standardized patients (SPs) or simulation program was also perceived as a weakness. The majority of our IPE activities have been paper-based cases. The use of simulation is gaining momentum as a model for teaching in IPE environment. This will provide the students with the opportunity to engage in different multi-professional settings before they start offering actual patient care. Furthermore, simulation facilities will also give students a positive learning experience in which they could refine their interprofessional skills in an environment that is free of risks which will ultimately reduce medical errors and improve patient outcomes [24].

The challenges that we are encountering are much like those faced by interprofessional educators elsewhere [24]. Implementing an IPE activity entails significant coordination in terms of scheduling, finding suitable space to accommodate students from different programs, and alignment and sequencing of topics in each respective curriculum. To help overcome the physical space challenge, QU CPH administration has considered IPE in the design of its new college building; however, this may not obviate QU campus restrictions by gender. Having flexible course schedules and a unified undergraduate IPE curricula across the different healthcare institutions in Qatar would help synchronization of IPE activities across institutions, yet requires organization and administrative support from all healthcare organizations and educational institutions in the country.

Another challenge considered by the faculty was low IPE “buy-in” among students that may translate into lack of enthusiasm or interest in participating in IPE activities. IPE necessitates collaboration not only from faculty members, but also among students from all participating healthcare disciplines. Qatar could consider having events or programs aimed at increasing the students’ awareness of the value and importance of IPE in terms of promoting team-based learning and potentially improving patient safety and healthcare delivery. Encouragingly, the first Interprofessional Education Student Society was created to facilitate interaction and collaboration between healthcare students in Qatar and enhance their learning environment. An additional concern was the mismatch between current state of collaborative practice care in Qatar and the IPE-related educational expectations. The goal of IPE is for students to ultimately work as part of an interprofessional team and apply the concepts and skills that they have acquired into practice, but the environment they will face upon graduation may preclude opportunities to interact with more than simply physicians and nurses [25].

In addition, a lack of structured and objective means to assess students was considered a significant shortcoming in the IPE activities, thus far. Currently, there are no standardized approaches for evaluating our IPE activities or for determining students’ achievement of the shared IPE competencies in pharmacy or even across the other healthcare disciplines in Qatar. Designing and implementing a comprehensive assessment plan that targets the goals and educational competencies of IPE with involvement from all healthcare training institutions is warranted [26]. Information regarding the impact of IPE on practice is another critical outcome measure lacking in ours and other IPE programming [23]. Indeed, despite healthcare educators’ investment in IPE, there is no strong evidence on its effectiveness on health processes and patient outcomes. A Cochrane review by Reeves et al. found some studies demonstrating the positive impact of IPE in areas such as diabetes care and emergency medicine, but other studies have failed to show IPE’s impact on professional practice or patient care [20]. It is not plausible at this stage to make any conclusions about IPE initiatives in Qatar and an association with patient outcomes, as we too need to design further studies in this regard.

## Conclusion

The development and implementation of IPE in a new setting results in many lessons learned that can benefit others attempting to develop or refine similar programming. First, it is useful to develop IPE competency domains that are relevant to all healthcare professions within the practice setting. Secondly, continuous professional development related to planning, facilitation, and assessment of IPE activities for new and current faculty is necessary to uphold the interprofessional integrity of the activity. Thirdly, coordinating schedules, devoting time, and allocating a dedicated space to conduct IPE activities are required to achieve active participation from all healthcare professions. Finally, disseminating one’s experiences with IPE activities through presentations and publications is essential to foster scholarship and collaboration among healthcare institutions. Furthermore, measuring the impact of the IPE initiatives is vital to ensure these endeavours are not futile in the healthcare setting.

## Abbreviation

IPE: Interprofessional education
